# Influence of Aminosilane Structure on 5-Fluorouracil Adsorption and Retention in Zeolites

**DOI:** 10.3390/ma19214485

**Published:** 2026-10-24

**Authors:** Ritsuko Sekiguchi-Arai, Masaya Komoto, Yoshiko Yamazaki, Naoki Noma, Kengo Oka, Mitsunobu Iwasaki

**Affiliations:** 1 Graduate School of Science and Engineering, Kindai University, 3-4-1 Kowakae, Higashiosaka 5778502, Osaka, Japan; 2633320239j@kindai.ac.jp; 2 Materials Research Department, SEED Co., Ltd., 1030-7 Fukuro, Konosu 3690131, Saitama, Japan; yoshiko_yamazaki@seed.co.jp; 3 Division of Joint Research Center, Kindai University, 3-4-1 Kowakae, Higashiosaka 5778502, Osaka, Japan; noma@apch.kindai.ac.jp; 4 Department of Applied Chemistry, Kindai University, 3-4-1 Kowakae, Higashiosaka 5778502, Osaka, Japan; koka@apch.kindai.ac.jp

**Keywords:** 5-fluorouracil, zeolite, aminosilane, adsorption, retention, ocular drug scavenging

## Abstract

**Highlights:**

- ZF showed the highest 5-FU adsorption among unmodified zeolites.
- Aminosilane functionalization enhanced 5-FU adsorption and retention.
- AEPMS produced the greatest adsorption improvement in several zeolites.
- APTMS- and APTES-functionalized ZF achieved ~98% 5-FU retention after 24 h.
- Amino-functionalized zeolites show promise as ocular 5-FU scavenging materials.

**Abstract:**

5-Fluorouracil (5-FU) can cause ocular adverse effects due to its presence in lacrimal fluid during treatment. To explore potential drug-scavenging materials for reducing excess 5-FU exposure, we investigated the adsorption and retention of 5-FU in zeolites and silane-functionalized zeolites. Seven commercial zeolites with different framework structures were evaluated for 5-FU adsorption from phosphate-buffered saline (PBS). Among the unmodified materials, Beta-type zeolite (ZF) exhibited the highest adsorption ratio (14.76%). To enhance drug-capturing performance, zeolites were functionalized with amino-containing silanes, including APTMS, APTES, AEPMS, and AHPMS. Functionalization altered both adsorption and retention behavior depending on the zeolite framework and silane structure. AEPMS increased 5-FU adsorption in several zeolites, whereas APTMS- and APTES-functionalized ZF exhibited the highest retention. After 24 h of immersion in PBS, the retention ratio increased from 7.63% for unmodified ZF to 98.47% and 98.25% for APTMS- and APTES-functionalized ZF, respectively. The silane structure that maximized adsorption differed from that conferring the highest retention, indicating distinct structure–property relationships. These results identify amino-functionalized zeolites as promising materials for capturing 5-FU and supporting their potential use as drug-scavenging materials in ophthalmic applications.

## 1. Introduction

Over the past decades, cancer has become a major global health challenge, with both incidence and mortality continuing to increase. The Global Burden of Disease (GBD) 2023 report estimated that 18.5 million individuals were newly diagnosed with cancer and 10.4 million died from the disease in 2023, with projections of 30.5 million new cases and 18.6 million deaths annually by 2050 [1]. Importantly, this growing disease burden has coincided with substantial improvements in patient survival, largely driven by advances in anticancer pharmacotherapy and multimodal treatment strategies. Consequently, clinical priorities have expanded beyond survival to include the management of treatment-related complications and the preservation of long-term quality of life.

**Figure 1:**
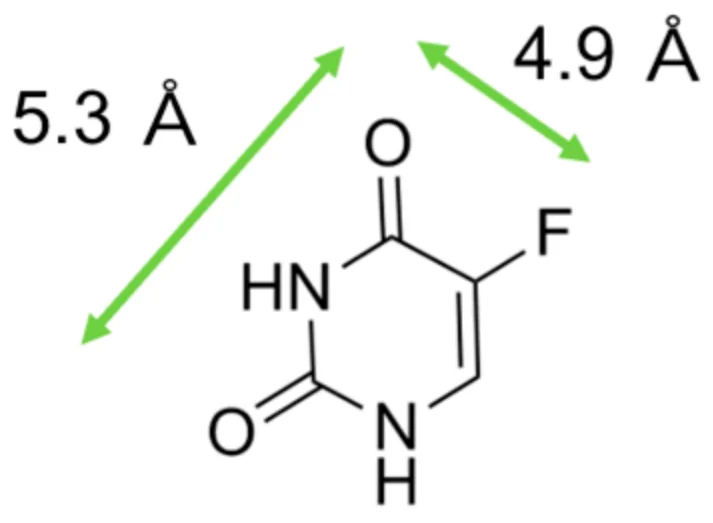
Chemical structure of 5-fluorouracil (5-FU).

Standard cancer therapies include surgery, radiotherapy, and chemotherapy, which are often combined to maximize therapeutic efficacy [2]. Among these approaches, chemotherapy remains central to systemic disease control. Chemotherapeutic agents are commonly classified based on their mechanisms of action, with antimetabolites representing a major class. These agents inhibit tumor cell proliferation by mimicking endogenous nucleic acid substrates, thereby disrupting DNA synthesis and cell cycle progression [3]. Tegafur, one of the first orally available anticancer agents, is a prodrug of 5-fluorouracil (5-FU; Figure 1), a widely used antimetabolite. Furthermore, TS-1, a combination formulation containing tegafur, is a standard treatment option for several malignancies, including gastric, colorectal, and pancreatic cancers [4,5].

**Figure 2:**
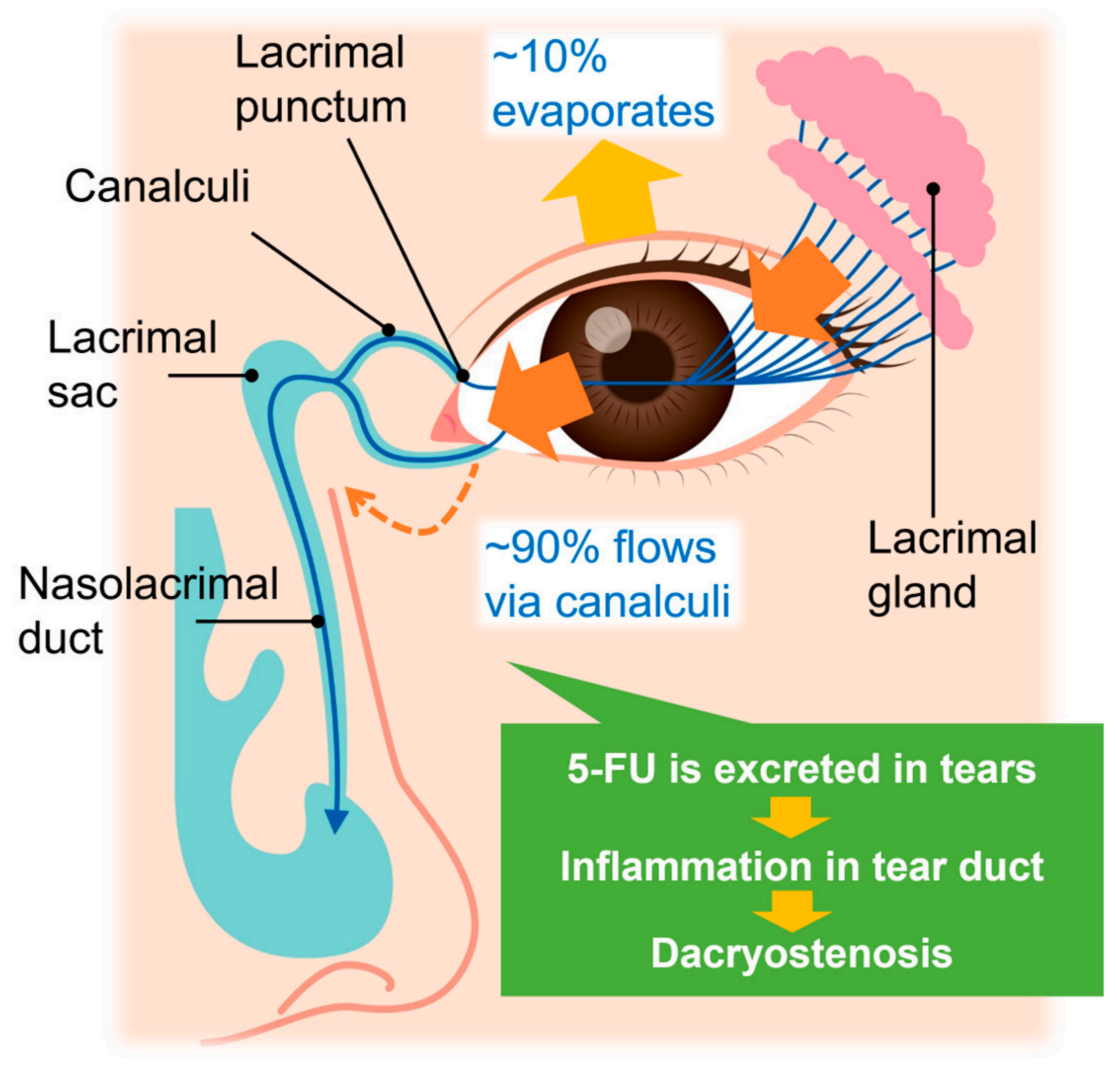
Proposed mechanisms of 5-fluorouracil-induced dacryostenosis. Adapted from Reference [10] under the CC BY 4.0 license.

Despite its therapeutic benefits, TS-1 is associated with adverse effects that can compromise patient well-being, including hematological toxicity, fatigue, gastrointestinal symptoms, and anorexia [6]. Beyond these systemic effects, ocular toxicities related to 5-FU exposure have received increasing attention. Prior studies demonstrated that 5-FU is transported into the lacrimal fluid, where it may contribute to lacrimal duct obstruction, corneal epithelial damage, and dry eye disease [5,6,7,8] (Figure 2). Corneal epitheliopathy has also been reported following topical 5-FU administration for ocular surface squamous neoplasia [9]. Although lacrimal irrigation with saline has been proposed to reduce local drug accumulation, its effectiveness remains unclear. This limitation may reflect continuous redistribution of 5-FU from the systemic circulation into lacrimal fluid, suggesting that local irrigation alone may not sufficiently prevent ocular toxicity. These observations underscore the need for alternative strategies that directly remove or sequester excess 5-FU in lacrimal fluid to mitigate drug-related ocular adverse effects.

If 5-fluorouracil (5-FU) can be scavenged from lacrimal fluid at the ocular surface, ocular exposure and the associated adverse effects may be reduced. We previously demonstrated that incorporating porous carbon black (CB) into a hydrogel increased the adsorption and retention of 5-FU [10]. However, CB has a broad, nonuniform pore-size distribution, and CB nanoparticles have been reported to adsorb multiple proteins in biological fluids [11]. Therefore, CB may also adsorb physiologically beneficial constituents of lacrimal fluid. In this study, we investigated zeolites with well-defined microporous structures as alternative adsorbents. We hypothesized that selecting a zeolite with pore dimensions matched to the molecular size of 5-FU would enable selective adsorption of 5-FU from lacrimal fluid and enhance its retention.

**Figure 3:**
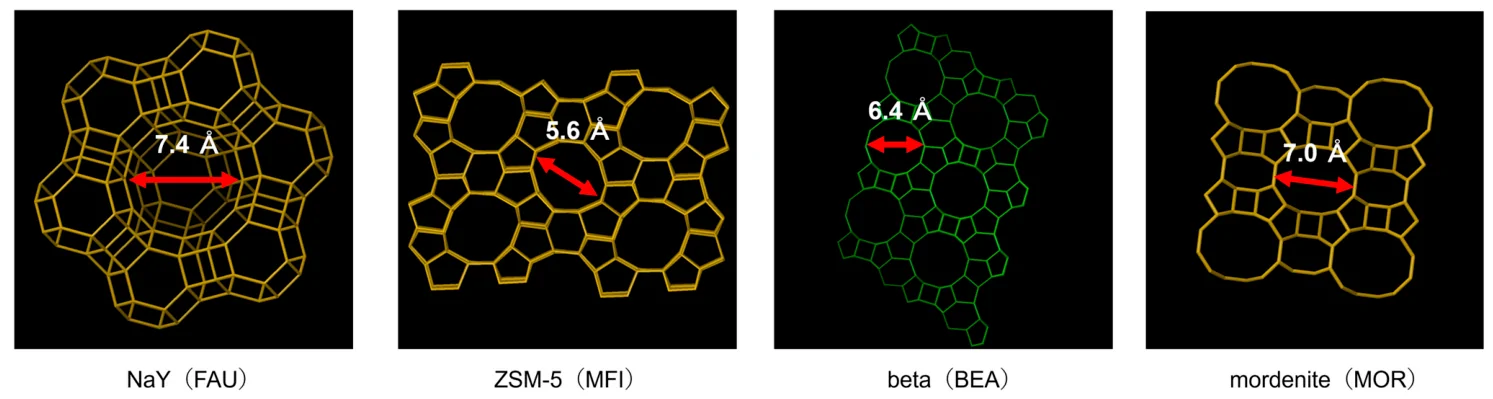
Representative framework structures of the zeolites evaluated in this study. Copyright © 2017 Structure Commission of the International Zeolite Association by Ch. Baerlocher, D. Brouwer, B. Marler, and L.B. McCusker, available at https://www.iza-structure.org/databases/ accessed on 1 October 2026 [13].

Zeolites are crystalline aluminosilicates composed of SiO_4_ and AlO_4_ tetrahedral units and are characterized by well-ordered microporous structures. More than 200 zeolite framework types with distinct pore sizes have been identified. Their adsorption behavior depends on pore size, the type of exchangeable cation, and the Si/Al ratio; notably, hydrophilicity and hydrophobicity can be tuned by adjusting the Si/Al ratio [12]. Representative framework structures of the zeolites evaluated in this study are shown in Figure 3.

Owing to these properties, zeolites are widely used in the chemical industry as adsorbents, molecular sieves, ion exchangers, and catalysts. They often exhibit superior selective adsorption compared with activated carbon and silica gel and are used in applications including natural gas purification, water treatment, and organic compound separation [14,15]. Faujasite-type zeolites can be synthesized without organic structure-directing agents, avoiding the additional high-temperature calcination step required to remove organic templates in template-assisted routes [16]. This is a potential environmental advantage of the synthesis route. Recent studies have investigated FAU- and BEA-type zeolites for ammonium removal [17] and FAU/CaCO_3_ composites for cadmium adsorption [18], illustrating the versatility of these materials as adsorbent platforms. Their biocompatibility and low toxicity have also driven growing interest in biomedical applications [18,19]. For example, in assays using Caco-2 cells, prolonged exposure to low concentrations of drug-free Beta zeolite did not significantly reduce cell growth [20], and NaX, NaY, and HY-type zeolites have been investigated as carriers for the antibiotic clofazimine [21]. In addition, nanozeolites A and Y showed low cytotoxicity under the in vitro conditions tested [22]. Zeolites with micropore sizes of approximately 3–8 Å are particularly suitable for adsorption of small-molecule drugs and have been extensively studied for sustained drug delivery systems (DDSs) and for encapsulation of poorly water-soluble drugs [23,24,25,26]. Surface functionalization (e.g., introduction of functional groups or polymer coatings) can alter surface chemistry and charge, thereby modulating drug adsorption behavior [27,28]. The introduction of amino groups onto the surfaces of carriers such as zeolites and silica has been reported to affect cytotoxicity and immune cell activation [29,30]. Organic surface modification has also been used to tailor zeolitic materials for the removal of other organic molecules. For example, Moraes et al. investigated myclobutanil adsorption using CTAB-functionalized zeolitic materials, including faujasite [31]. This example supports the use of surface chemistry as a design variable for organic-molecule capture.

Although many studies have examined the uptake and sustained release of 5-FU from zeolites [20,32], they have largely emphasized drug release in vivo, whereas studies specifically targeting drug capture and adsorption remain limited. Accordingly, we evaluated 5-FU adsorption and retention for several unmodified zeolites and for zeolites functionalized with silane coupling agents. We specifically examined how zeolite framework structure and aminosilane architecture influenced uptake from phosphate-buffered saline (PBS) and retention during subsequent immersion in PBS.

## 2. Materials and Methods

### 2.1. Materials

5-Fluorouracil was purchased from Tokyo Chemical Industry Co., Ltd. (Tokyo, Japan). Dulbecco’s phosphate-buffered saline (PBS; calcium- and magnesium-free, pH = 7.4) was purchased from FUJIFILM Wako Pure Chemical Corporation (Osaka, Japan). Seven commercial zeolites (FUJIFILM Wako Pure Chemical) were designated ZA–ZG; their framework types and supplier-reported properties are summarized in Table 1.

**Figure 4:**
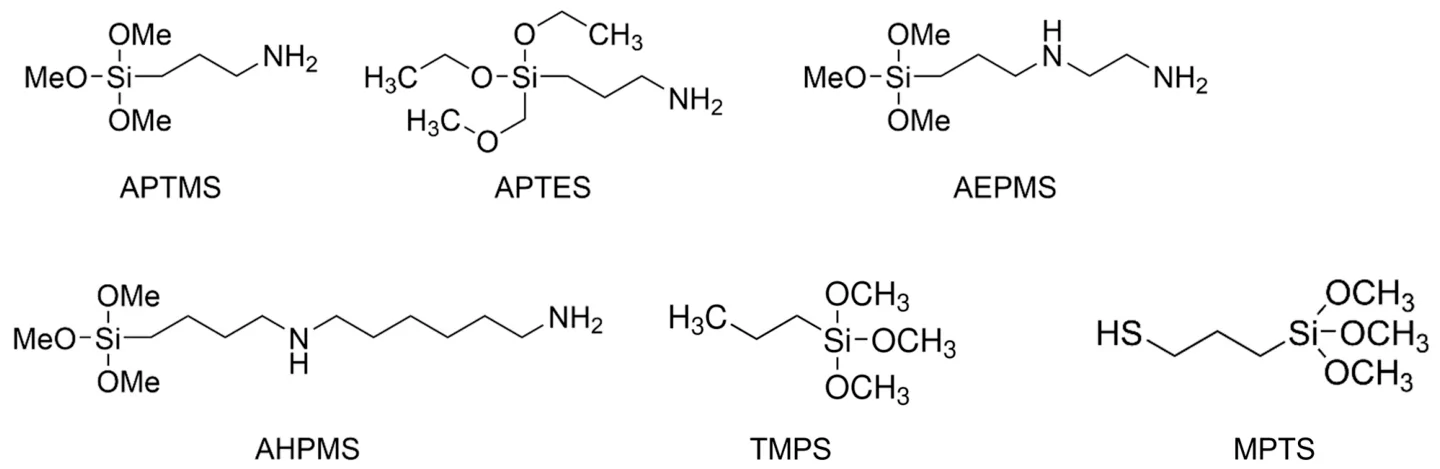
Chemical structures of the functional silane coupling agents used in this study.

The amino-functional silanes were 3-aminopropyltrimethoxysilane (APTMS), 3-aminopropyltriethoxysilane (APTES), N-(2-aminoethyl)-3-aminopropyltrimethoxysilane (AEPMS), and N-[3-(trimethoxysilyl)propyl]hexane-1,6-diamine (AHPMS) (Figure 4). Trimethoxy(propyl)silane (TMPS) and (3-mercaptopropyl)trimethoxysilane (MPTS) were used to evaluate non-amino surface functionalities. AEPMS was purchased from Shin-Etsu Chemical (Tokyo, Japan), and all other functional silanes were purchased from Tokyo Chemical Industry (Tokyo, Japan).

### 2.2. Surface Functionalization

Each zeolite was dried at 110 °C overnight. The dried zeolite (100 mg) was dispersed in 10 mL of 10% (*v*/*v*) ethanol in water containing 0.50, 1.0, 2.0, 3.0, or 5.0 wt% silane and stirred at room temperature for 0.5, 2, 4, 8, or 24 h. After the reaction, the solid was washed twice with deionized water and twice with ethanol, collected by centrifugation at 4000 rpm for 20 min, and dried at 80 °C overnight.

**Figure 5:**
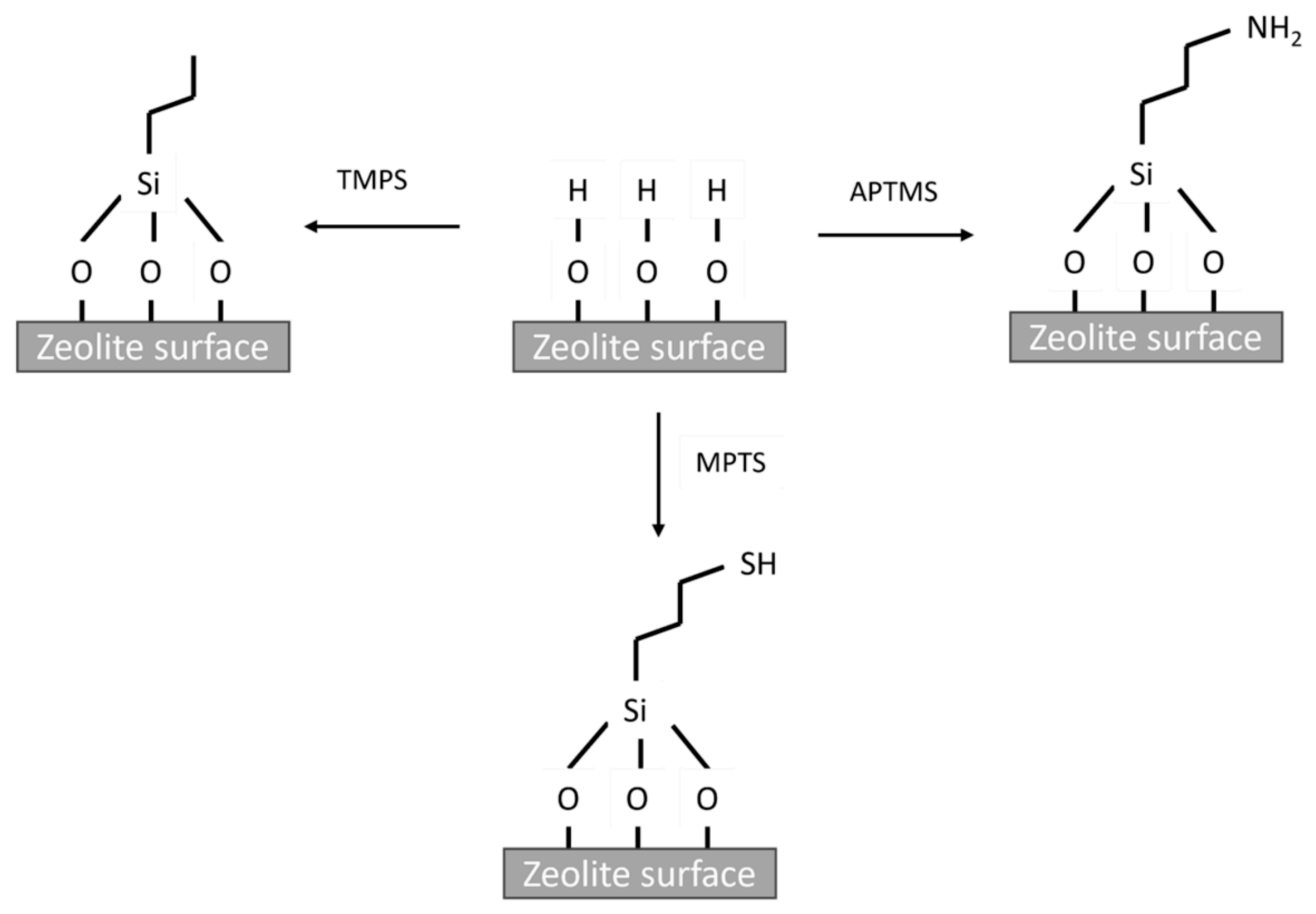
Schematic illustration of silane modification on the zeolite surface.

APTMS, APTES, AEPMS, and AHPMS were evaluated as amino-functional modifiers. In the concentration-dependence experiments, the AEPMS concentration was varied for ZC, ZD, ZF, and ZG; in all other comparative adsorption experiments, silane functionalization was performed using 2.0 wt% silane for 24 h. TMPS (2.0 wt%) was introduced using the same procedure to generate propyl-functionalized surfaces, and MPTS (2.0 wt%) was used analogously to generate mercapto-functionalized surfaces. The proposed surface functionalization mechanism is illustrated in Figure 5.

### 2.3. Characterization

Nitrogen adsorption measurements were performed using a TriStar 3000 analyzer (Shimadzu Corporation, Kyoto, Japan). Samples were predried at 120 °C for 1 h under a low pressure below 100 mTorr. Specific surface areas were calculated by the Brunauer–Emmett–Teller method using six data points in the relative-pressure range P/P_0_ = 0.05–0.30. Total pore volume was estimated from nitrogen uptake at P/P_0_ = 0.995.

Fourier-transform infrared (FTIR) spectra were recorded at room temperature using an FT/IR-4100 spectrometer (JASCO Corporation, Tokyo, Japan). The surface potential of all zeolite samples was measured using a Zetasizer (ELSZ-2000, OTSUKA ELECTRONICS Co., LTD, Osaka, Japan) after dispersing each sample in water; measurements were performed at pH 7. Thermogravimetric analysis (TG-DTA) was conducted using a simultaneous thermal analyzer (Thermo plus EVO TG8120, Rigaku Co., Tokyo, Japan) with Al_2_O_3_ as the reference material under an air atmosphere. The sample (10 mg) was placed in a platinum pan and heated from 27.1 to 800 °C at 10 °C min^−1^. For functionalized samples, the blank-pan baseline was subtracted from the resulting weight-loss profiles.

### 2.4. 5-FU Adsorption

Each sample (50 mg) was dispersed in 4 mL of 100 ppm 5-FU in PBS and agitated at 250 rpm at room temperature for 24 h. This contact time was selected based on a previous study [33]. The suspension was filtered through a cellulose acetate membrane, and the equilibrium 5-FU concentration was determined at 266 nm using a UV–visible spectrophotometer (UV–vis: NanoDrop2000, Thermo Fisher Scientific K.K., Tokyo, Japan). PBS exhibited negligible background absorbance in the ultraviolet region (Figure S1) [34]. The adsorption ratio was calculated as follows:

(1) $$5-\mathrm{FU}\;\mathrm{adsorption}\;\mathrm{ratio}\;(\%)=\;\frac{\left(C_{0}\;-\;C_{e}\right)}{C_{0}}\times 100\%$$

where *C*_0_ and *C_e_* are the initial and equilibrium concentrations of 5-FU, respectively. Unless otherwise stated, values are reported as the mean ± standard deviation from three independent measurements (*n* = 3). The calibration curve covered the concentration range of 10 to 200 ppm (Figure S2).

### 2.5. 5-FU Release and Retention

After 24 h of adsorption, zeolite particles were recovered by ultrafiltration and vacuum-dried at room temperature for 24 h. Each 5-FU-loaded zeolite sample (50 mg) was then dispersed in 4 mL of fresh PBS and agitated at room temperature. At predetermined time points (0.5, 1, 2, 3, and 24 h), 50 μL aliquots of the supernatant were collected, and the 5-FU concentration was measured by UV-vis spectroscopy. PBS was not replaced after sampling. The retention ratio was calculated as follows:

(2) $$5-\mathrm{FU}\;\mathrm{retention}\;\mathrm{ratio}\;(\%)=\;\frac{\left(C_{s}\;-\;C_{r}\right)}{C_{s}}\times 100\%$$

where *C_s_* is the initial concentration of adsorbed 5-FU and *C_r_* is the concentration of 5-FU released into PBS at the specified time. The retained amount per unit volume (*q_v_*) was obtained as follows:

*q_v_* = *q_ret_*ρ (3)

where *q_ret_* is the retained amount per unit mass, and ρ is the density of the carrier. For ZF, a literature reference density of 1.61 g cm^−3^, previously assumed for zeolite Beta characterization, was adopted [35] and was assumed to be unchanged by functionalization. This value represents a reference conversion rather than a measured bulk or tapped density in PBS at the specified time.

### 2.6. Statistical Analyses

All data are expressed as mean ± SD (*n* = 3). Statistical analyses were performed using one-way or two-way ANOVA, as appropriate, followed by Tukey’s multiple comparison test. A value of *p* < 0.05 was considered statistically significant.

## 3. Results and Discussion

### 3.1. Framework Properties and Adsorption

Figure S3 shows the UV–Vis spectra of the 100 ppm 5-FU solution before and after adsorption by the zeolites, and Table 2 summarizes the corresponding 5-FU adsorption ratios. Among the unmodified zeolites, ZF exhibited the highest 5-FU adsorption ratio of 14.76% (1.20 mg g^−1^). The value in parentheses represents the mass of 5-FU adsorbed per gram of zeolite.

Among the ZSM-5 samples (ZA–ZC), ZC had the lowest Si/Al ratio and the highest adsorption ratio. This trend suggests that framework composition influences 5-FU uptake, potentially through differences in framework polarity, surface hydroxyl density, and competition with water [20].

ZG and ZD were both NaY-type materials with similar Si/Al ratios; however, ZG had the larger supplier-reported surface area and the higher adsorption ratio. This comparison suggests that accessible surface area contributed to uptake. ZE showed the lowest adsorption ratio; its one-dimensional mordenite channels may limit molecular diffusion relative to the three-dimensional pore systems of NaY and Beta zeolites. Guest loading in faujasites is known to depend jointly on pore accessibility, framework composition, and adsorbate–framework interactions [36].

Furthermore, the ammonium form of ZF should be considered as a potential contributor to its high 5-FU uptake. Whereas all zeolites other than ZF were in the sodium form, ZF was in the ammonium form. However, although ZC and ZF exhibit broadly similar physicochemical properties, and the amount of 5-FU adsorbed by ZF was approximately twice that adsorbed by ZC. This difference may reflect the identity of the counter-cation (NH_4_^+^ versus Na^+^). Its NH_4_^+^ counterions may contribute favorably to drug–host associations by acting as N–H hydrogen-bond donors toward the carbonyl oxygen atoms of 5-FU. This interaction is chemically plausible as a mechanism for stabilizing adsorbed 5-FU and may complement the adsorption environment provided by the Beta framework. Syrový et al. reported hydrogen bonding and electrostatic association between ammonium-related N–H species and oxygen-containing functional groups, including carbonyl groups, in β-ammonium zeolite/cellulose acetate composites [37]. However, this study did not evaluate the contribution of NH_4_^+^ independently of framework structure and other factors. Further investigation, including comparisons of samples with the same Beta framework and Si/Al ratio but different counter-cations, is needed to clarify the effect of NH_4_^+^ on 5-FU adsorption and retention. Recent work on H-Beta likewise indicates that the Si/Al ratio, drug loading, molecular crowding, and intermolecular interactions can jointly modulate 5-FU behavior [38]. Together, these findings suggest that ZF—with a relatively low Si/Al ratio (~20), small pore size (~6 Å), large specific surface area (~600 m^2^ g^−1^), and ammonium counter-ions—exhibited the highest adsorption capacity.

#### Retention by Unmodified Zeolites

**Figure 6:**
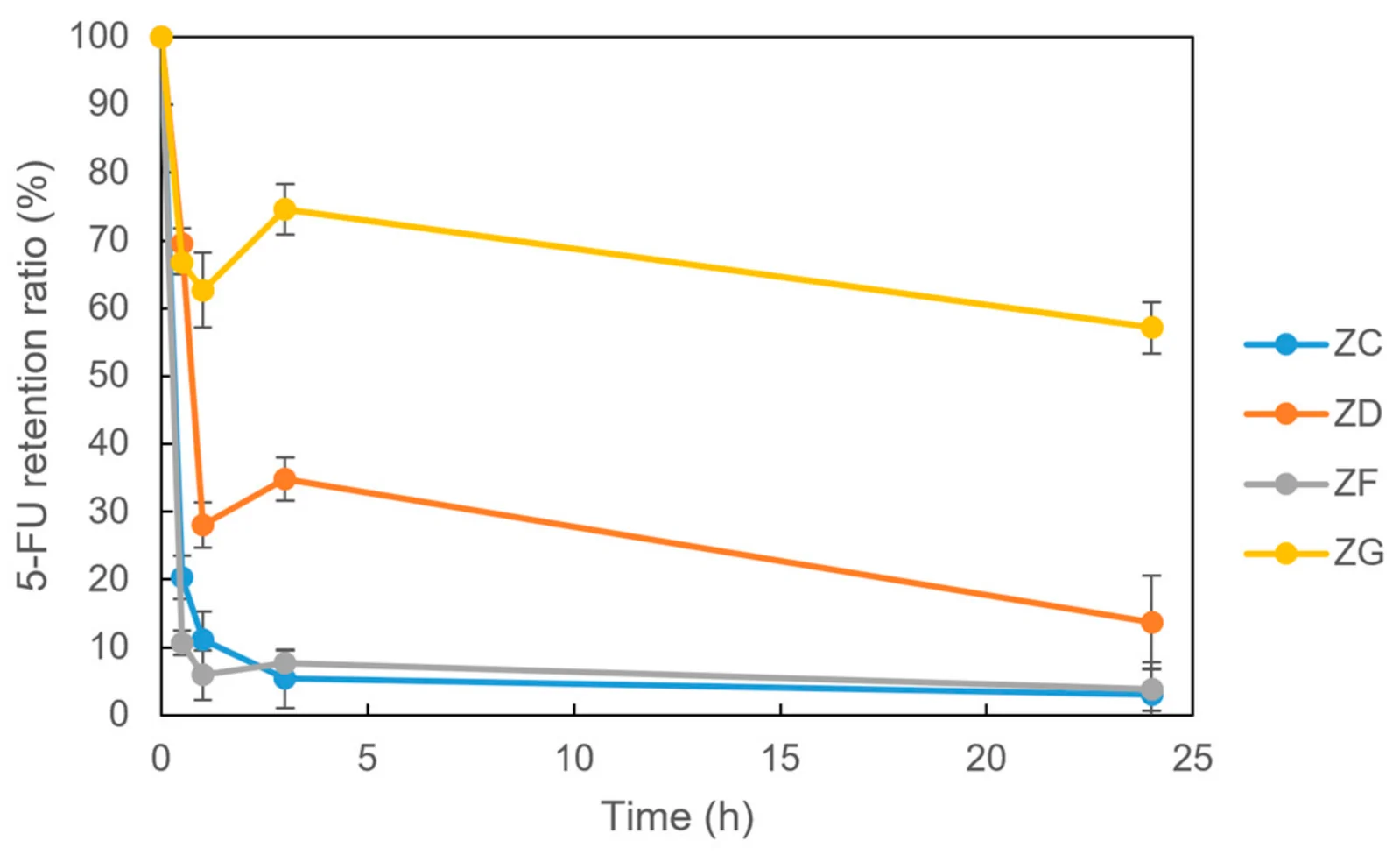
Time-dependent 5-FU retention profiles of unmodified zeolites (pH = 7.4). Data are presented as mean ± standard deviation (n = 3).

The physicochemical properties of zeolites are listed in Table S1. Figure 6 and Figure S4 show the retention profiles of 5-FU-loaded, unmodified zeolites during immersion in PBS. Retention decreased over time for all samples, consistent with desorption driven by the concentration gradient and competition with the aqueous phase. Similar effects of zeolite composition on 5-FU loading and release have been reported for HY zeolites [39].

Among the four zeolites, ZG exhibited particularly high 5-FU retention. ZG and ZD differed from the other two zeolites primarily in pore size and Si/Al ratio; notably, the Si/Al ratios of the other two zeolites were substantially higher than those of ZG and ZD. In zeolites with high Si/Al ratios, the lower framework Al content and fewer charge-compensating cations reduce the framework negative-charge density and polarity. Consequently, electrostatic interactions and hydrogen bonding with 5-FU are weakened, limiting immobilization after adsorption and promoting 5-FU desorption during immersion in PBS [39]. Although ZG and ZD had nearly identical Si/Al ratios and pore sizes, the specific surface area of ZG was approximately 1.17-fold higher than that of ZD. This difference likely contributes, at least in part, to the higher retention of ZG: its large surface area (467 m^2^ g^−1^) provides more adsorption sites for 5-FU, potentially leading to stronger adsorption and slower desorption.

Spanakis et al. reported stronger van der Waals interactions and slower 5-FU diffusion in BEA than in NaX–FAU [20], supporting the role of the framework structure in modulating drug release. After 24 h, ZG retained approximately 0.40 mg of 5-FU per gram of carrier, whereas ZC, ZD, and ZF retained approximately 0.02–0.05 mg g^−1^. On a carrier-volume basis, ZG retained approximately 0.56 mg cm^−3^, comparable to the approximately 0.5 mg cm^−3^ reported for CB in the source comparison [10].

### 3.2. Characterization of Functionalized Zeolites

ZC, ZD, ZF, and ZG were selected for functionalization because they represent distinct framework types and adsorption behaviors. Table 3 compares the specific surface areas and total pore volumes before and after APTMS treatment.

The measured surface areas of the unmodified samples were lower than the supplier-reported values in Table 1, which may reflect differences in pretreatment, residual moisture, crystallinity, or particle aggregation. APTMS treatment decreased both surface area and total pore volume for all zeolites. The largest decrease was observed for ZC, whose surface area decreased from 91.3 to 7.65 m^2^ g^−1^ (a 91.6% reduction). This pronounced decline is consistent with reduced pore accessibility after modification; similar decreases after aminosilane treatment have been reported for Y-type zeolite [40].

**Figure 7:**
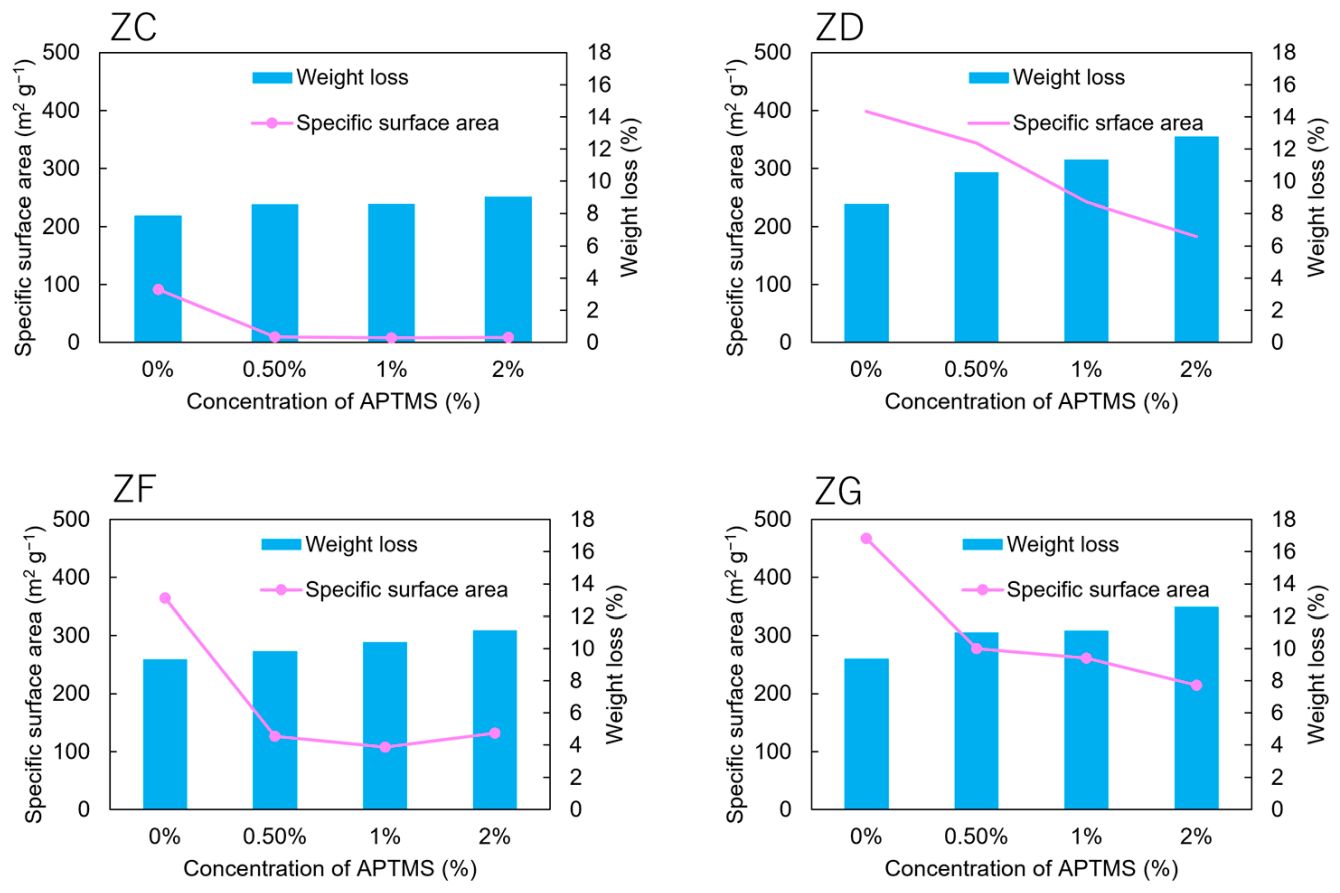
Specific surface areas and thermogravimetric weight losses of APTMS-functionalized zeolites prepared at different APTMS concentrations.

Figure 7 summarizes the specific surface areas and thermogravimetric mass losses of the functionalized zeolites. The mass losses attributed to silane-derived organic components indicate an estimated organic content of approximately 0.5–3 wt%, depending on the material and functionalization conditions. For ZD, ZF, and ZG, the mass loss increased with increasing APTMS concentration, whereas ZC showed a weaker concentration dependence. These TGA-based estimates provide an indication of organic incorporation but should not be equated with grafting density, surface coverage, or accessible amino-group density.

Amino-functionalization shifted the zeta potentials of all four zeolites from negative to positive values (Table 4). These positive shifts are consistent with protonatable amino groups at the particle–water interface.

TMPS- and MPTS-functionalized ZF remained negatively charged and were slightly more negative than unmodified ZF (Table 5). These findings indicate that the zeta-potential changes reflect successful grafting of the functional groups onto the zeolite surface [41].

**Figure 8:**
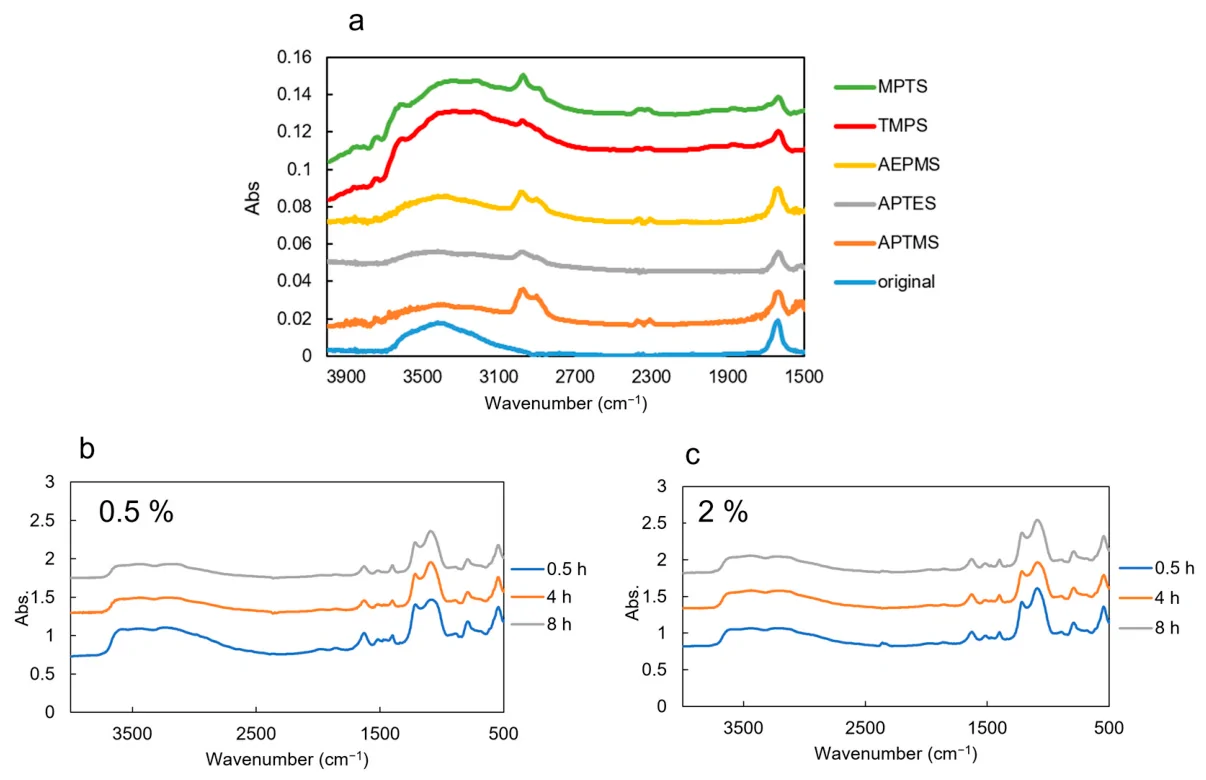
FTIR spectra of (**a**) unmodified and silane-functionalized ZF samples; (**b**,**c**) ZC-APTMS samples prepared at different reaction times and APTMS concentrations.

In the FTIR spectra of the functionalized ZF samples (Figure 8a), bands near 2958 and 2865 cm^−1^ were assigned to aliphatic C–H stretching vibrations. In the amino-functionalized samples, a band near 1529 cm^−1^ was consistent with N–H bending [42]. Together, these features support the presence of silane-derived organic moieties on the zeolite surface. The S–H vibration of MPTS was not clearly resolved under the present measurement conditions.

Based on previous studies of zeolite silanization [40,43], the present functionalization is primarily interpreted as the formation of an amino-bearing organic layer on the external zeolite surfaces and near pore entrances. The C–H and N–H bands support the presence of silane-derived organic groups, while positive zeta-potential shifts are consistent with amino functionalities at the particle–water interface. In this model, externally accessible amino groups can provide interaction sites for 5-FU, whereas deposition near pore entrances can reduce pore accessibility and affect adsorption and subsequent retention. The decrease in nitrogen-accessible surface area is consistent with partial obstruction of pore entrances and does not necessarily require extensive deposition within the internal micropores. The importance of host surface properties and pore structure for drug–host interactions has also been illustrated for clofazimine-loaded silica materials and zeolites [21].

### 3.3. 5-FU Adsorption by Functionalized Zeolites

**Figure 9:**
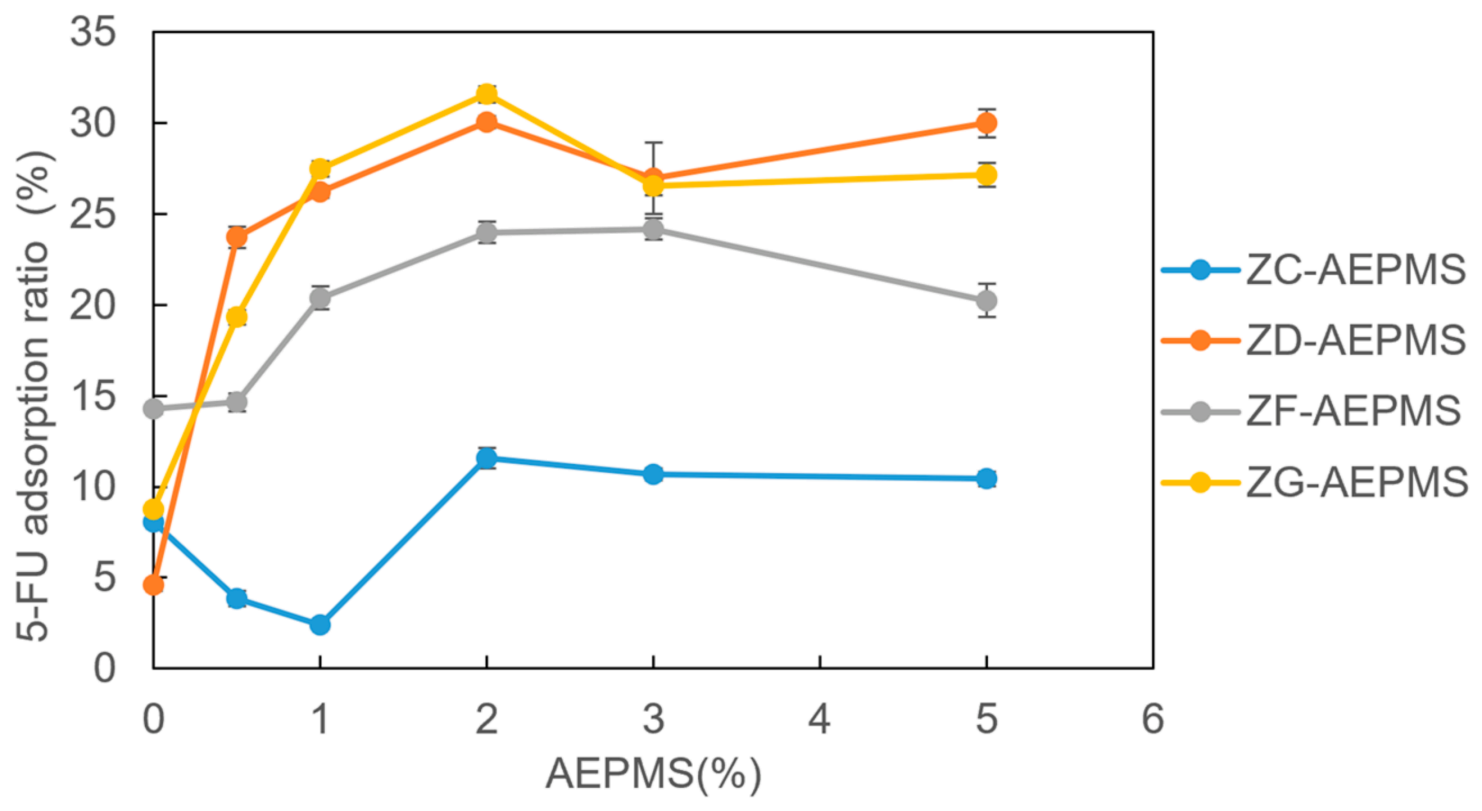
5-FU adsorption ratios of AEPMS-functionalized zeolites prepared at different AEPMS concentrations after 24 h of functionalization (pH = 7.4). Data are presented as mean ± standard deviation (n = 3).

Figure 9 shows the concentration dependence of 5-FU adsorption for AEPMS-functionalized ZC, ZD, ZF, and ZG after 24 h of functionalization. ZD-AEPMS, ZF-AEPMS, and ZG-AEPMS adsorbed more 5-FU than their corresponding unmodified zeolites, whereas ZC showed no clear improvement. Across all four zeolites, 2 wt% AEPMS yielded the highest measured adsorption ratio among the tested concentrations.

Silane deposition in aqueous media proceeds through hydrolysis, surface condensation, and, in some cases, silane self-condensation; the resulting layer is governed by water content, pH, silane concentration, reaction time, and the nature of the oxide surface [44,45,46]. At high AEPMS concentrations, self-condensation or multilayer formation may reduce access to zeolite pores or amino groups.

**Figure 10:**
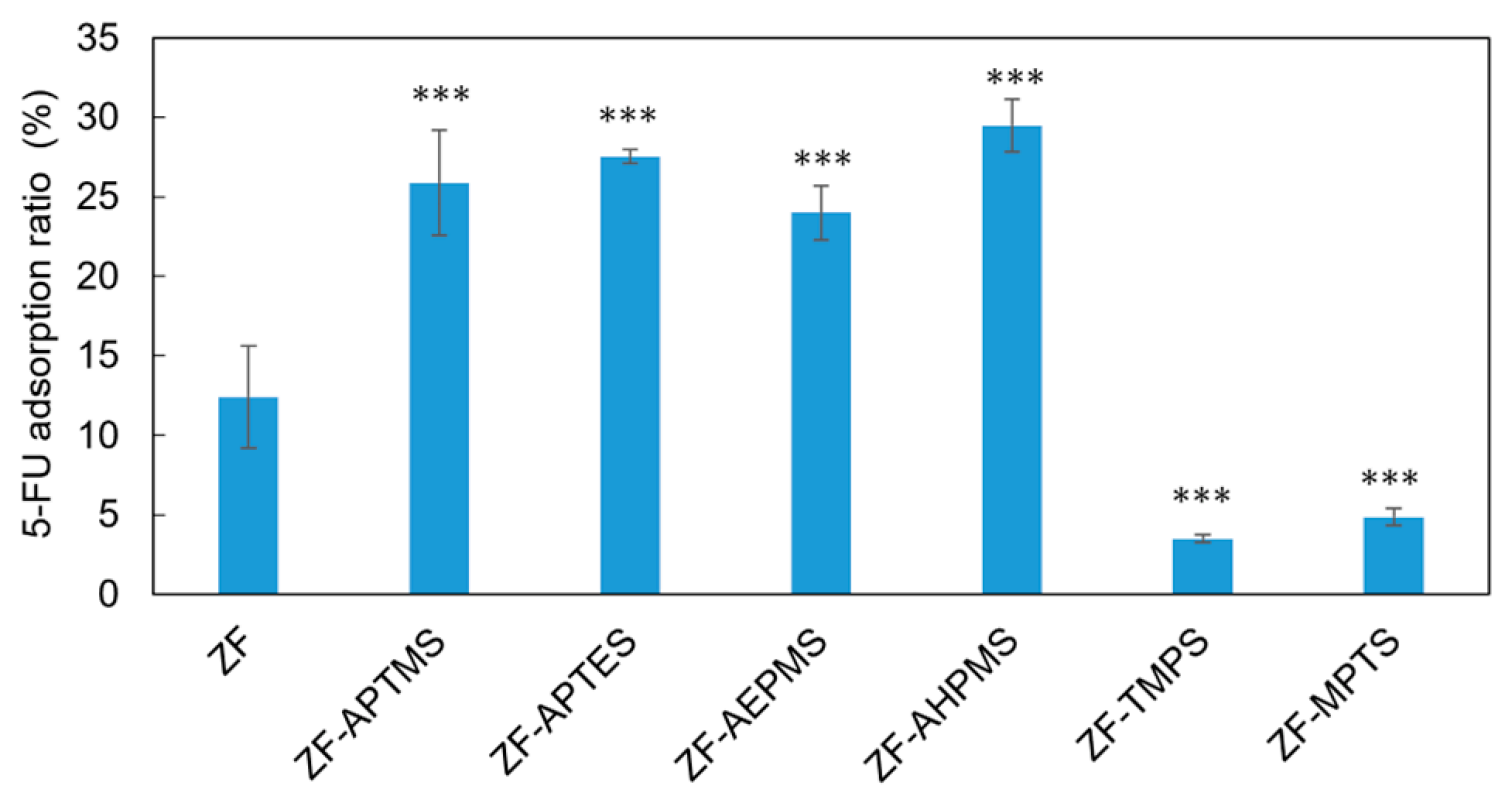
5-FU adsorption ratios of unmodified and silane-functionalized ZF samples (pH = 7.4). Data are presented as mean ± SD (n = 3). Statistical analysis was performed using one-way ANOVA followed by Tukey’s multiple comparison test. *** *p* < 0.001 versus ZF.

Figure 10 compares the adsorption ratios of unmodified ZF with those of ZF functionalized with 2 wt% APTMS, APTES, AEPMS, AHPMS, TMPS, or MPTS. Aminosilane functionalization markedly enhanced 5-FU adsorption by ZF, with ZF-APTMS and ZF-APTES exhibiting adsorption ratios of approximately 25.8% (2.03 mg g^−1^) and 27.5% (2.11 mg g^−1^), respectively, whereas the TMPS- and MPTS-functionalized samples exhibited lower adsorption capacities than unmodified ZF. Because 5-FU contains carbonyl and imide groups, it can readily form hydrogen bonds with amino functionalities. By contrast, propyl and mercapto groups are expected to interact less strongly with 5-FU and thus provide limited adsorption enhancement. Furthermore, functionalization can decrease the effective pore size and partially block pore entrances, thereby limiting the diffusion of 5-FU into the zeolite framework. These effects likely account for the reduced adsorption capacities of the TMPS- and MPTS-functionalized samples relative to the unmodified zeolite. Overall, these results indicate that introducing amino groups is an effective strategy to enhance 5-FU adsorption; therefore, subsequent investigations focused on amino-containing silane coupling agents.

**Figure 11:**
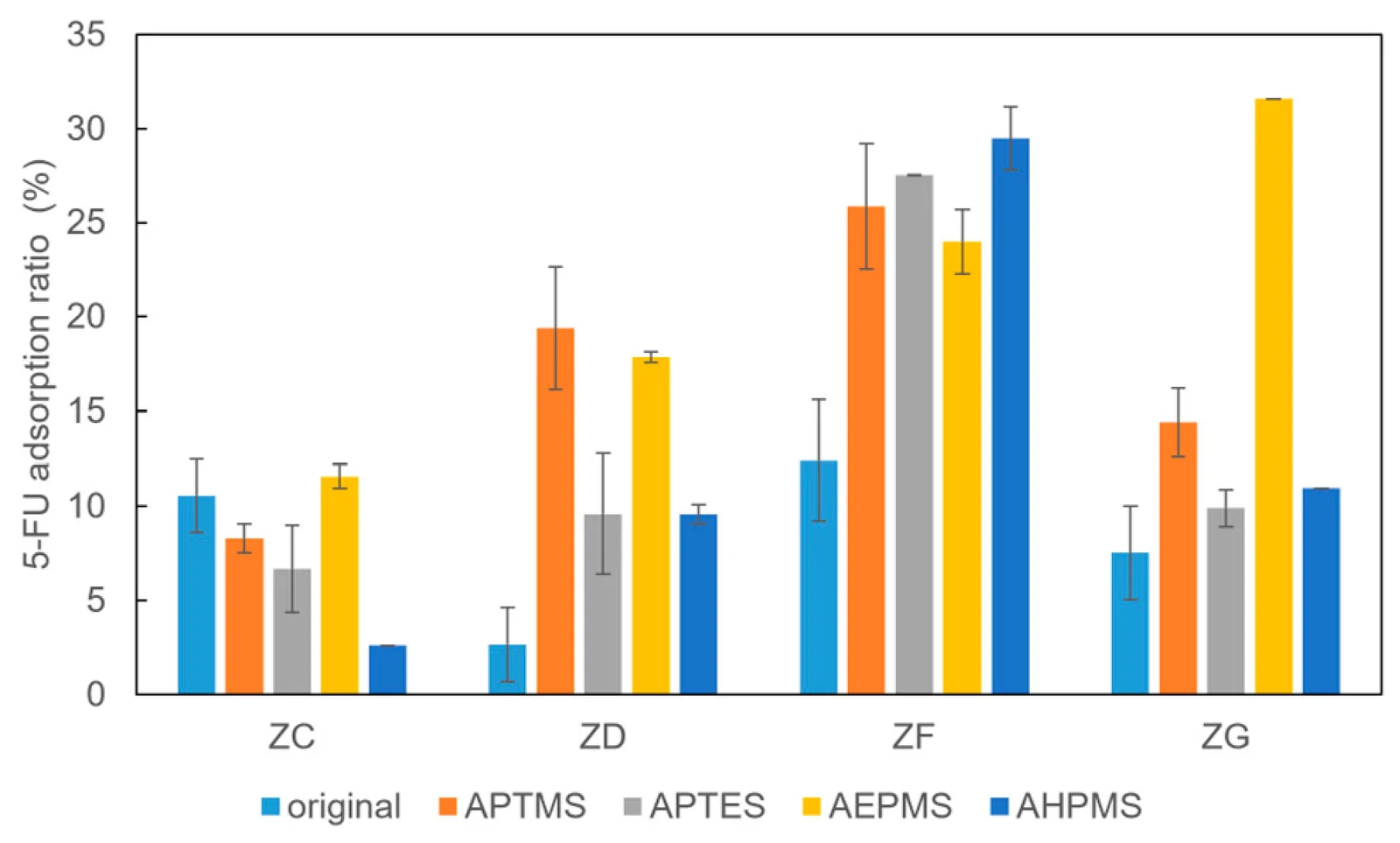
5-FU adsorption ratios of four zeolites functionalized with different silanes (pH = 7.4). Data are presented as mean ± SD (n = 3). Statistical analysis was performed using two-way ANOVA, with zeolite type and silane modification as factors. Significant effects of zeolite type, silane modification, and their interaction were observed (all *p* < 0.05).

The combined comparison across zeolites and silane modifiers (Figure 11) further indicated that the optimal modifier depended on the host zeolite. Overall, AEPMS functionalization produced the greatest improvement in 5-FU adsorption. This enhancement suggests a key contribution from hydrogen bonding between the silane-introduced amino groups and the carbonyl and imide groups of 5-FU.

**Figure 12:**
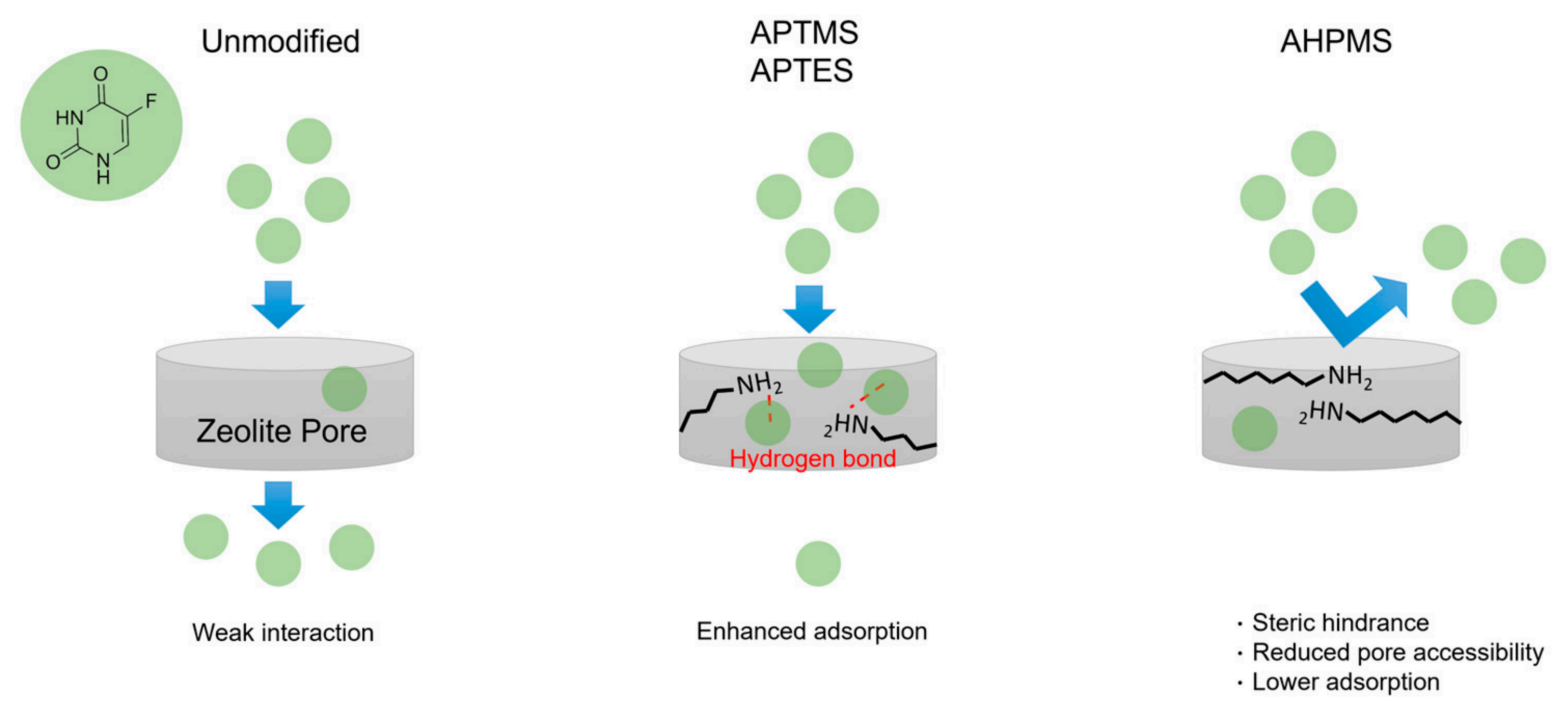
Proposed adsorption mechanism of 5-FU on amino-functionalized zeolites.

For ZD and ZG, which have relatively large pore structures, the AEPMS-functionalized samples exhibited the highest adsorption ratios among the tested silanes. This trend suggests that amino groups introduced on the external surface or near pore entrances provided additional interaction sites for 5-FU, thereby promoting hydrogen bonding and increasing adsorption. In contrast, adsorption decreased after AHPMS functionalization, suggesting that 5-FU uptake was hindered by features associated with the silane structure, including steric effects and increased diffusion resistance within the pore network (Figure 12).

By contrast, amino functionalization did not yield a significant adsorption improvement for ZC. As indicated by the nitrogen adsorption and TG-DTA results, ZC initially possessed a pore structure favorable for 5-FU adsorption. However, functionalization likely reduced the effective pore diameter and partially blocked pore entrances, thereby limiting diffusion into the pore system and reducing adsorption within the pores. Similar behavior has been reported for other drug adsorption systems [47]. Although amino functionalization can strengthen hydrogen-bonding and electrostatic interactions with adsorbates, excessive surface modification can decrease effective pore size and restrict pore accessibility, ultimately diminishing adsorption performance [48].

The observed adsorption trends are consistent with a balance between the affinity introduced by amino groups and changes in pore accessibility. Accessible amino groups may form hydrogen bonds with the carbonyl and N-H groups of 5-FU, while surface hydroxyl groups may also contribute. Protonated amino groups may additionally interact with the anionic fraction of 5-FU. With a reported pKa of approximately 8.0 [49], 5-FU is predominantly neutral at pH 7.4, so adsorption should be considered in terms of both speciation and hydration. The positive zeta-potential shifts measured in water at pH 7 support a change in interfacial charge after functionalization. Their relevance to adsorption in PBS must account for electrolyte screening and possible competition from phosphate species. The reduction in nitrogen-accessible surface area is consistent with altered pore accessibility, although it does not directly quantify the accessibility of 5-FU in aqueous solution. These observations support a proposed interaction model whose individual contributions remain to be determined.

Although amino functionalization improved the adsorption ratio of ZF, no pronounced differences were observed among the amino silanes tested, with adsorption ratios remaining within a narrow range of 24–29%.

### 3.4. 5-FU Retention by Functionalized ZF

**Figure 13:**
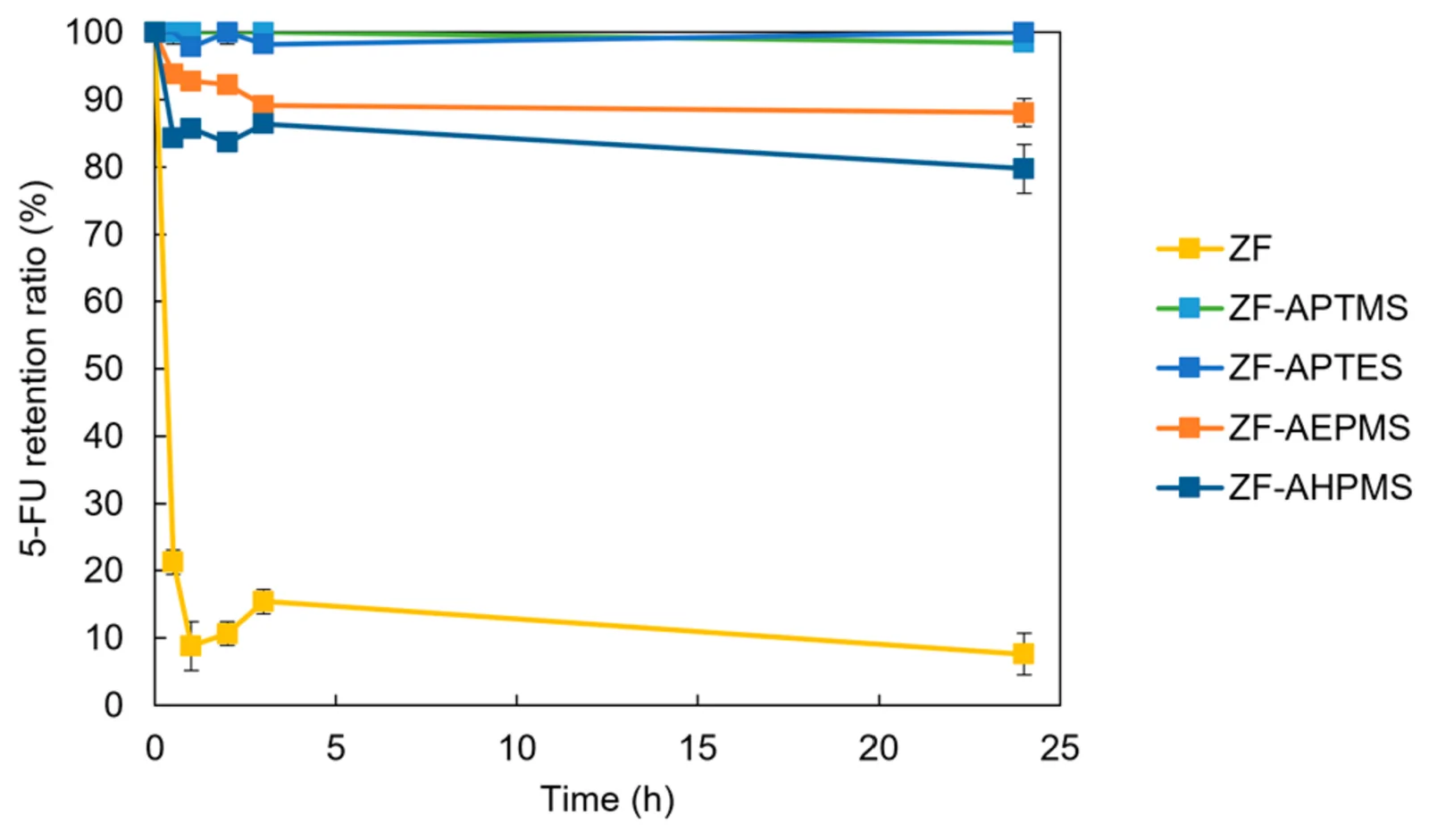
Time-dependent 5-FU retention profiles of unmodified and amino-functionalized ZF samples (pH = 7.4). Data are presented as mean ± standard deviation (n = 3).

Figure 13 and Table 6 present the time-dependent retention profiles and 24 h retention ratios for unmodified and amino-functionalized ZF. Additional data are provided in Table S2. APTMS and APTES yielded the highest 24 h retention ratios (98.47% and 98.25%, respectively), followed by AEPMS (88.09%) and AHPMS (79.73%). In contrast, unmodified ZF retained only 7.63% of the initially adsorbed 5-FU after 24 h.

The higher retention observed for APTMS- and APTES-functionalized ZF may reflect differences in the spatial presentation of amino groups. APTMS and APTES share the compact –(CH_2_)_3_–NH_2_ functionality. In AEPMS and AHPMS, the corresponding surface-proximal amine is secondary and carries an additional aminoethyl or aminohexyl substituent, respectively. These substituents change the steric environment around the surface-proximal amine and increase conformational freedom. Studies of amine-functionalized silica demonstrate that linker geometry, steric hindrance, and interactions with surface silanols can influence the availability and function of amine sites [50]. By analogy, within the proposed external-surface modification model, the compact aminopropyl groups may permit less hindered contact between 5-FU and binding sites near the zeolite interface. The additional flexible chains of AEPMS and AHPMS may alter amino-group orientation through folding or association with nearby surface groups, reducing the availability of effective binding sites despite their larger nominal amino-group count.

Notably, ZF-AHPMS exhibited the most positive measured zeta potential in water among the amino-functionalized ZF samples (Table 4), but the lowest 24 h retention ratio among them (Table 6). Thus, the zeta-potential values measured in water do not alone explain the retention ranking in PBS. Local hydration may also contribute; the importance of interfacial organization and hydration in aqueous media is illustrated by studies of zwitterionic–polyester composite films [51]. Because contact angles and accessible amino-group densities were not independently measured, the proposed structural explanation remains to be verified.

After 24 h of immersion in fresh PBS, approximately 1%, 25%, and 27% of the total 5-FU initially present in the adsorption solution were estimated to remain in unmodified ZF, ZF-APTMS, and ZF-APTES, respectively. Notably, the retention ratios of ZF-APTMS and ZF-APTES were comparable to those of CB (25.2%). However, the amount of 5-FU retained per unit carrier volume was substantially higher for ZF-APTMS and ZF-APTES (approximately 3 mg cm^−3^) than for CB (approximately 0.4 mg cm^−3^), indicating a markedly greater retention capacity for the modified zeolites. Given that the pore size of ZF is 6–7 Å, 5-FU can be efficiently accommodated within the zeolite pores. In addition, the amino-containing functional groups on the surface may interact with 5-FU, thereby strengthening post-adsorption retention.

The proposed contact lens design confines the zeolite particles to the peripheral region, leaving the central optical zone particle-free to preserve its transparency. Our previous study demonstrated CB-printed hydrogels with a transparent optical zone and largely preserved storage moduli in the HG56 formulations [10]. These findings support the feasibility of incorporating adsorbent particles while maintaining a clear central zone and useful mechanical properties. Nevertheless, optical performance and mechanical properties must be verified at the selected loading of amino-functionalized zeolites. The particle-concentration-dependent rheology reported by Sun et al. [52] also supports considering particle loading and dispersion during formulation development. Spanakis et al. [20] demonstrated framework-dependent differences in loading and release between NaX–FAU and BEA. Dunkley et al. [38] reported that approximately 98% and 93% of the loaded 5-FU were released from H-Beta with Si/Al ratios of 19 and 180, respectively, after 120 min in PBS at pH 7.4. In the present study, the 24 h retention ratio in PBS increased from 7.63% for unmodified ZF to 98.47% and 98.25% for ZF-APTMS and ZF-APTES, respectively. However, differences in loading solvent, initial drug concentration, drug loading, release-medium volume, sample form, and observation time preclude a direct ranking of material performance across these studies. The contribution of the present work is the evaluation of how aminosilane functionalization affects both adsorption and subsequent retention under the same experimental conditions, providing complementary criteria for selecting materials for 5-FU scavenging.

### 3.5. Limitations and Future Perspectives

These results support the potential of amino-functionalized zeolites as 5-FU-scavenging materials; however, the present study has several limitations. First, all adsorption and retention experiments were performed in PBS as a simplified model system. Thus, the selective adsorption of 5-FU in tear fluid and the effects on beneficial tear components remain unverified. Future studies should evaluate the selectivity of 5-FU adsorption in artificial lacrimal fluid containing proteins, lipids, and competing solutes, as well as the effects of zeolite dosage and contact time on adsorption and subsequent release kinetics. In addition, the ocular biocompatibility of the materials, including cytotoxicity and irritation potential, should be investigated using relevant ocular cell lines and in vivo models. Further studies are also required to evaluate the long-term stability of the amino-functionalized zeolites under physiological conditions. For practical ophthalmic applications, ocular anatomical and physiological barriers, as well as the transparency, water content, oxygen permeability, and mechanical properties of zeolite-containing disposable contact lens materials, should be assessed. Future studies should also assess the scalability of the functionalization process, the reproducibility of silane incorporation, manufacturing costs, and the potential for regeneration and reuse of zeolite materials. These investigations will be essential for translating the present findings into clinically applicable ocular drug-scavenging systems.

## 4. Conclusions

Aminosilane functionalization altered 5-FU adsorption and retention in a manner dependent on both the zeolite and the silane structure. Among the unmodified materials, ZF exhibited the highest adsorption ratio (14.76%). Functionalization with APTMS or APTES yielded 24 h retention ratios of 98.47% and 98.25%, respectively, compared with 7.63% for unmodified ZF, corresponding to increases of 90.84 and 90.62 percentage points, respectively. ZF functionalized with AEPMS and AHPMS retained 88.09% and 79.73%, respectively.

These findings demonstrate that amino-functionalized zeolites can achieve excellent 5-FU adsorption and retention. The performance demonstrated in this study suggests that functionalized zeolites may be promising drug-scavenging materials for ophthalmic applications. In future work, these functionalized zeolites will be incorporated into hydrogel contact lens materials to mitigate drug-related side effects associated with ocular drug administration. By selectively adsorbing excess drug from lacrimal fluid, these zeolite-containing hydrogels may provide a strategy to improve the safety of ophthalmic therapies while maintaining therapeutic efficacy.

## Figures and Tables

**Table 1:** Physicochemical properties of the zeolites used in this study.

Sample	Framework	Pore Size (Å)	Surface Area (m^2^ g^−1^)	Si/Al
**ZA**	ZSM-5	5–6	432	60
**ZB**	ZSM-5	5–6	464	90
**ZC**	ZSM-5	5–6	430	22.2
**ZD**	NaY	9	660	5.6
**ZE**	Mordenite	7	360	17.8
**ZF**	Beta-ammonium	6–7	669	24.3
**ZG**	NaY	9	927	5.16

**Table 2:** 5-FU adsorption ratios of unmodified zeolites. Data are presented as mean ± standard deviation (n = 3).

Sample	Adsorption Ratio (%)
**ZA**	2.61 ± 0.20
**ZB**	2.27 ± 0.79
**ZC**	8.07 ± 1.92
**ZD**	4.59 ± 1.96
**ZE**	1.36 ± 0.27
**ZF**	14.76 ± 3.18
**ZG**	8.76 ± 2.50

**Table 3:** Specific surface areas and total pore volumes of unmodified and APTMS-functionalized zeolites.

Sample	Specific Surface Area (m^2^ g^−1^)	Total Pore Volume (cm^3^ g^−1^)
**ZC**	91.3	0.098
**ZC-APTMS**	7.65	0.035
**ZD**	398	0.28
**ZD-APTMS**	242	0.17
**ZF**	364	0.61
**ZF-APTMS**	108	0.50
**ZG**	467	0.23
**ZG-APTMS**	262	0.15

**Table 4:** Zeta potentials (mV) of unmodified and amino-functionalized zeolites (pH = 7).

Sample	Unmodified	APTMS	APTES	AEPMS	AHPMS
**ZC**	−49.2	52.9	41.0	7.1	53.7
**ZD**	−23.4	21.6	25.7	5.5	56.7
**ZF**	−22.8	27.6	27.3	4.6	31.8
**ZG**	−30.3	31.9	18.8	11.6	29.0

**Table 5:** Zeta potentials (mV) of unmodified, TMPS-functionalized, and MPTS-functionalized ZF (pH = 7).

Sample	Unmodified	TMPS	MPTS
**ZF**	−22.8	−27.7	−29.8

**Table 6:** 5-FU retention ratios of ZF samples after 24 h in PBS. Data are presented as mean ± standard deviation (n = 3).

Sample	5-FU Retention Ratio at 24 h (%)
**ZF**	7.63 ± 3.11
**ZF-APTMS**	98.47 ± 0.17
**ZF-APTES**	98.25 ± 0.32
**ZF-AEPMS**	88.09 ± 0.21
**ZF-AHPMS**	79.73 ± 0.35

## Data Availability

The datasets presented in this article are not readily available because they are being used in an ongoing study. Requests to access the datasets should be directed to the corresponding authors.

## Supplementary Materials

The following supporting information can be downloaded at https://www.mdpi.com/article/10.3390/ma19214485/s1, Figure S1: UV-vis spectrum of PBS; Figure S2: Calibration curve for 5-FU at 266nm; Figure S3: UV-vis spectrum of 5-FU, ZC and ZF (pH = 7.4); Figure S4: UV-Vis spectrum of PBS during 5-FU release from ZC (pH = 7.4); Table S1: Time-Dependent Release of 5-FU from Zeolites into PBS (ppm); Table S2: Time-Dependent Release of 5-FU from Functionalized Zeolites into PBS (ppm).
