# Supplementary material for: Influence of Aminosilane Structure on 5-Fluorouracil Adsorption and Retention in Zeolites
Source: Materials (Basel). 2026 Oct 24;19(21):4485. doi: 10.3390/ma19214485 (PMC13764206; doi:10.3390/ma19214485)
Supplement: Supplementary file 1 [file materials-19-04485-s001.zip › materials-4601626-supplementary.pdf]

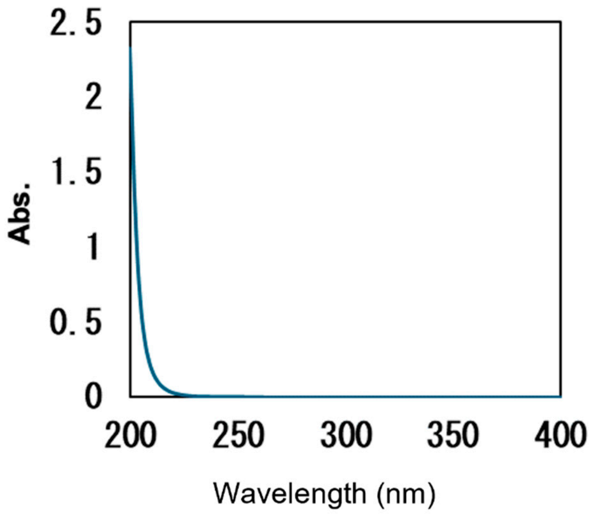

Figure S1. UV-vis spectrum of PBS.

As shown in Figure S1, PBS showed negligible absorbance in the ultraviolet region.

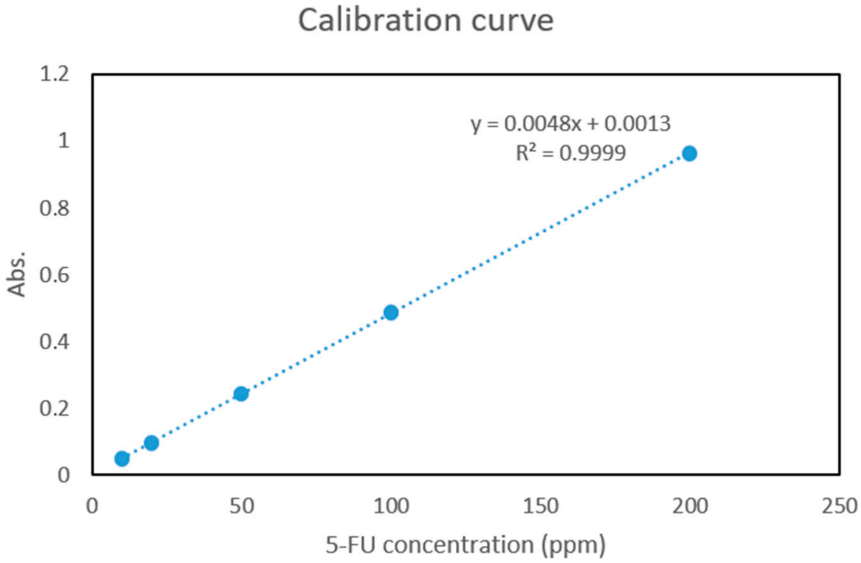

Figure S2. Calibration curve for 5-FU at 266nm.

Figure S2 shows the calibration curve for 5-FU at 266 nm. The calibration curve covered the concentration range of 10 to 200 ppm. PBS was used as the blank control.

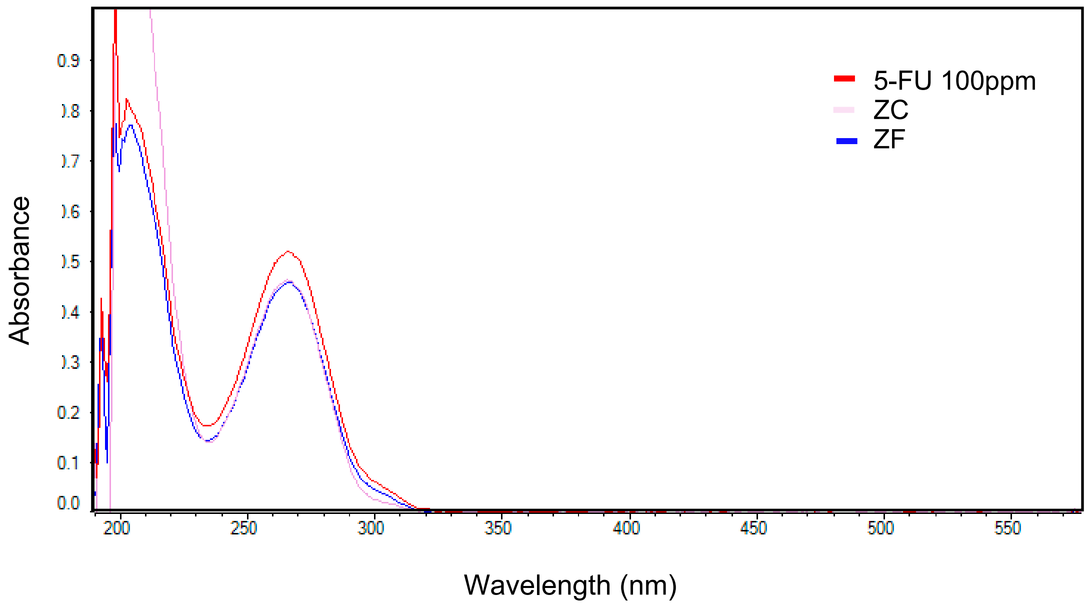

Figure S3. UV-vis spectrum of 5-FU, ZC and ZF (pH=7.4)

The characteristic absorption peak of 5-FU was observed at 266 nm. The absorbance decreased in the presence of ZC and ZF, suggesting that the concentration of 5-FU in PBS was reduced due to adsorption onto the zeolite samples.

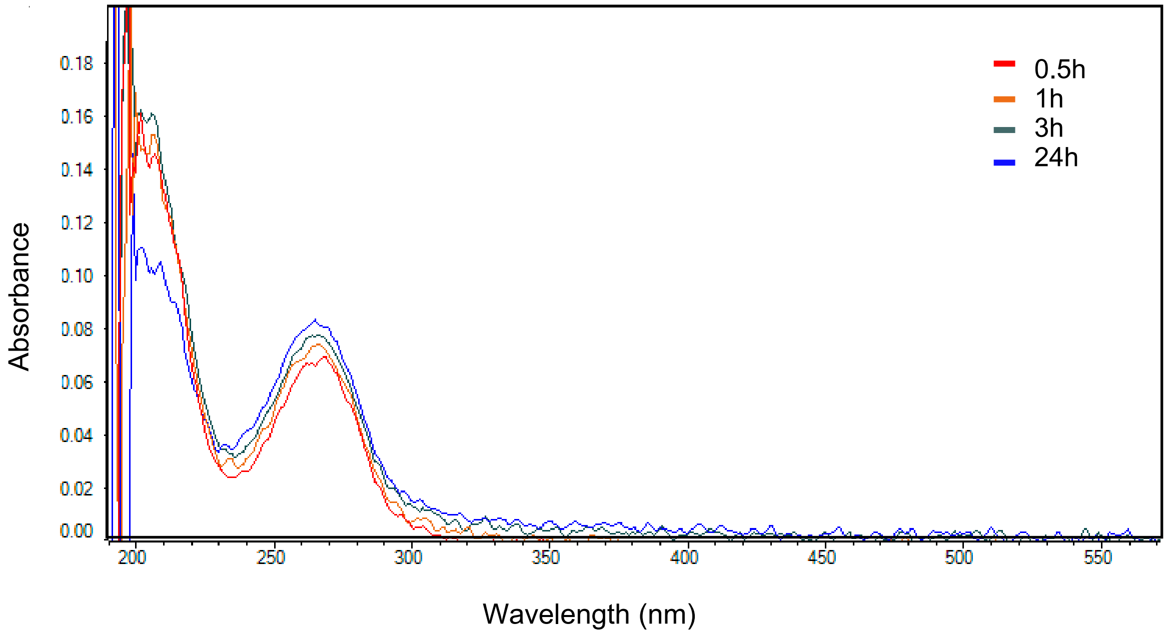

Figure S4. UV-Vis spectrum of PBS during 5-FU release from ZC (pH=7.4).

Figure S4 shows the UV-Vis spectrum of PBS following the replacement of the 5-FU-

adsorbed ZC. The concentration of 5-FU in PBS increased over time, indicating the gradual release of adsorbed 5-FU from the zeolite. Similar trends were observed for ZD, ZF, and ZG.

Table S1. Time-Dependent Release of 5-FU from Zeolites into PBS (ppm)

	ZC	ZD	ZF	ZG
Time (h)	5-FU concentration (ppm)			
0	11.57	0.47	17.68	5.05
0.5	6.01	0.62	15.69	1.80
1	6.77	2.00	15.20	0.85
2	7.46	1.73	17.07	0.07
3	8.16	1.38	17.68	1.52
24	8.02	3.32	15.97	0.97

Table S2. Time-Dependent Release of 5-FU from Functionalized Zeolites into PBS (ppm)

	ZF	ZF-APTMS	ZF-APTES	ZF-AEPMS	ZF-AHPMS
Time (h)	concentration (ppm)				
0	17.68	18.52	15.76	17.02	30.09
0.5	15.69	18.52	15.76	13.61	25.38
1	15.20	18.11	15.41	13.13	25.79
2	17.07	18.11	15.48	12.02	25.17
3	17.68	18.32	15.34	12.30	24.00
24	15.97	18.25	15.55	12.92	26.00
